# The Trend of Bacterial Nanocellulose Research Published in the Science Citation Index Expanded From 2005 to 2020: A Bibliometric Analysis

**DOI:** 10.3389/fbioe.2021.795341

**Published:** 2022-01-17

**Authors:** Yuh-Shan Ho, A. F. M. Fahad Halim, Mohammad Tajul Islam

**Affiliations:** ^1^ Trend Research Centre, Asia University, Taichung, Taiwan; ^2^ Department of Textile Engineering, Primeasia University, Dhaka, Bangladesh; ^3^ Department of Textile Engineering, Ahsanullah University of Science and Technology, Dhaka, Bangladesh

**Keywords:** bacterial nanocellulose, front page, SCI-EXPANDED, scientometrics, *TC*_*year*_, *C*_*year*_, *CPP*_*year*_

## Abstract

To gain insight into the trend of bacterial nanocellulose research, a bibliometric analysis was performed using the Science Citation Index Expanded database from 2005 to 2020. The study concentrated on the publication’s performance in terms of annual outputs and citations, mainstream journals, categories of the Web of Sciences, leading countries, prominent institutions, and trends in research. Current research priorities and future trends were analyzed after summarizing the most commonly used keywords extracted from words in the paper title analysis, authors’ keyword analysis, and *KeyWords Plus*. The findings revealed that the annual output in the form of scholarly articles on bacterial nanocellulose research steadily increased during the first quartile of the study period, followed by a very rapid increase in the last five-years of the study. Increasing mechanical strength would remain the main future focus of bacterial nanocellulose research to create its scope in different field of applications.

## 1 Introduction

The substance, which is made up of 99% water along with 1% high molecular and highly crystalline polymer with a distinct molecular and supramolecular pattern, is an uncommon yet intriguing challenge in polymer research and application. This explanation applies precisely to particular cellulose with nanostructures, and it is provided here in the form of nanocellulose. Polysaccharide cellulose is a highly significant and intriguing biopolymer, as well as a nearly endless raw source for sustainable polymers. The worldwide reawakening of multidisciplinary cellulose investigation and the application of this plentiful organic polymer has been fueled by the trend of renewable resources and the trend of creating novel goods for science, health, and technology in the last decade (Klemm et al., 2005).

The cellulose polymer is particularly noteworthy because of its distinct structure, which differs significantly from that of typical manmade polymers. This hydrophilic, chiral, biodegradable, and chemically modifiable linear hard-chain homopolymer is made up of repeated connections of glucose building units. The vast hydrogen bond connection that creates the semi-crystalline fiber shape is likewise based on this chemical structure. As a result, supramolecular organization and specific assembly, which are influenced by cellulose supply and processing, play a major role in cellulose characteristics (Klemm et al., 2005).

In this context, obtaining diverse forms of cellulose with numerous sources, supramolecular geometries, unique characteristics, and varying availability is highly essential in order to extend the use of cellulose, comprising the advance of unique materials with breakthrough novel functionalities, and also broaden the scope of the application. Floras are the most common source of cellulosic material. Cotton seed hairs have cellulose that is nearly pure. Lignocellulose, on the other hand, forms natural complexes with lignin as well as other carbohydrates, which are then removed from them using wide-ranging pulping process which are chemical, isolation, and purifying procedures. Still wood pulp (Koch, 2006) is the utmost significant raw material for the manufacture of thin sheets and cardboards, cellulose regenerated fibers, and cellulose esters and ether palettes on a large scale. Photosensitive films, adsorption medium and construction constituents, boring technologies, different surface coatings, medicines, food and cosmetics additives all employ these compounds as significant and well-known active components. The biocompatibility and chirality of cellulose are also used in a variety of novel applications.

In addition to plants, cellulose is produced by bacteria, algae, and fungus. *Acetobacter* strains—reclassified as *Gluconobacter*—are particularly suited for cellulose production among the cellulose-forming bacteria. They are not harmful, can be cultivated in the lab, and are often found in fruits and fruit products (Andritsou et al., 2018). The cell-free system might be a viable alternative to the existing microbial cellulose synthesis method constraints (Ullah et al., 2015). The enzymatic process involved in the normal biochemical route of cellulose formation by microbial cells can be carried out *in vitro* to produce cell-free cellulose. When compared to a whole-cell system, such a system will offer a number of benefits, including longer and continuous production due to immobilization, sustainable and efficient cofactor renewal, and the prevention of aberrant buildup of intermediate metabolites (Khattak et al., 2014; Khan et al., 2015). Hestrin and Schramm created a medium for the synthesis of bacterial cellulose (BC) in 1954, and it quickly became popular among scientists and manufacturers (Hestrin and Schramm 1954). Nevertheless, in recent times, numerous fermentation characteristics, for instance pH control and carbon sources have been researched in attempt to reduce the cost of producing this biopolymer (Huang et al., 2016; Tyagi and Suresh 2016; Costa et al., 2018). The bacterium *K. xylinus* was employed as the major source of carbon glycerol in biodiesel by Vazquez et al. (2013). They got a polymer yield of 10 g/L following 14 days of static culture. Kurosumi et al. employed orange juice as a polymerization catalyst and obtained 5.9 g/L of BC after incubation for 14 days (Kurosumi et al., 2009).

All bacterial cellulose must be nanocellulose because the bacteria that make it can only bind fibrils on the nanometer scale. BC is eco-friendly for a variety of reasons, including its high purity, which needs less energy to purify than plant cellulose (Klemm et al., 2005). The microbes may circulate freely in the media or adhere to the cellulose fibres throughout the fermentation procedure, resulting in a highly swollen gel structure (Dufresne et al., 1997). BC has the same chemical construction as plant-derived cellulose. Nevertheless, BC has the benefit of containing no lignin, pectin, hemicellulose, or other biological constituents involved with the formation of plant cell walls (Jonas and Farah 1998; Iqbal et al., 2014). BC offers a wide variety of uses due to its purity and unique physical and chemical features (Svensson et al., 2005; Czaja et al., 2006; Shah et al., 2013; Silva et al., 2014). Gao, Qiuying et al. prepared regenerated BC fibers by dissolving BC in N-methylmorpholine-N-oxide monohydrate (Gao et al., 2011). Wang, Li et al. used cellulose dissolution and physical and chemical crosslinking techniques to create a novel regenerated bacterial cellulose/polypyrrole/carbon nanotube electroactive hydrogel that promotes cell proliferation and wound healing through an electric field (Wang, Li. et al., 2020) As a result of the foregoing qualities, BC might be a good choice for developing innovative products.

The natural substance BC was originally used in a dessert which is free of calorie known as Nata de Coco, which is now a popular Asian dish. BC has the same molecular formula as plant derived cellulose, with the exception of foreign groups generated by the processing of plant cellulose in the latter. However, the prominent structural features and attributes of BC that are critical to its practical application differ significantly from those of wood cellulose: high purity, great degree of polymerization (DP) having good crystallinity, better water content, and excellent mechanical consistency. The biosynthetic synthesis of BC, as described later, and the consequent particular supramolecular structure cause these specific characteristics. There is no suitable composite partner for a nanofiber network generated during the self-assembly of cellulose molecules in an aqueous solution for example, wood biosynthesis (Klemm et al., 2005). These polymers are called nanocellulose if they are made up of nanofibers and the nanofiber structure dictates the product attributes. BC containing cellulose at nanoscale is known as bacterial nanocellulose (BNC).

The term nano-sized cellulose is used to describe secluded crystallites and whiskers generated by acid-catalyzed cellulose breakdown. This subject, as well as the use of nanocellulose in composite materials, has been extensively researched (Azizi Samir et al., 2005; Ljungberg et al., 2005). Furthermore, the same term has recently been applied to a low concentration trimethylsilyl cellulose solution and consequent diversified desilylation of the foremost produced silyl cellulose layer during cellulose regeneration as an open Spin-coated section of a tiny cellulose patch (Kontturi et al., 2005). In recent years, there has been an increase in global involvement in the areas of substantial scientific research and practical application of nanocellulose.

Bibliometrics is a valuable method for mapping the literature on a specific research topic, and it has been utilized to track the research trend in specialized fields of study recently, such as metal-organic frameworks (Wang and Ho, 2011), drinking water research (Fu et al., 2012), and risk assessment (Mao et al., 2010). Bibliometrics is a research methodology based on quantitative analysis and statistics commonly used in library and information sciences. This research method can reveal the distribution patterns of articles published in the database within a given topic, field, institution, and country. The Science Citation Index Expanded (SCI-EXPANDED) from the Web of Science Core Collection of the Clarivate Analytics (previously known as Thomson Reuters) is the most valuable and widely used data repository for analyzing scientific achievements across all fields of research.

As previously stated, a variety of nanocellulose is produced directly by the biosynthesis of specific bacteria. It is necessary to create complex biosynthesis/biotechnology processing and large-scale production in order to produce a very pure product with the crucial qualities. Another type of nanocellulose can be made employing a controlled mechanical decomposition step to achieve favorable product features from a practically endless source of raw wood.

**FIGURE 1:**
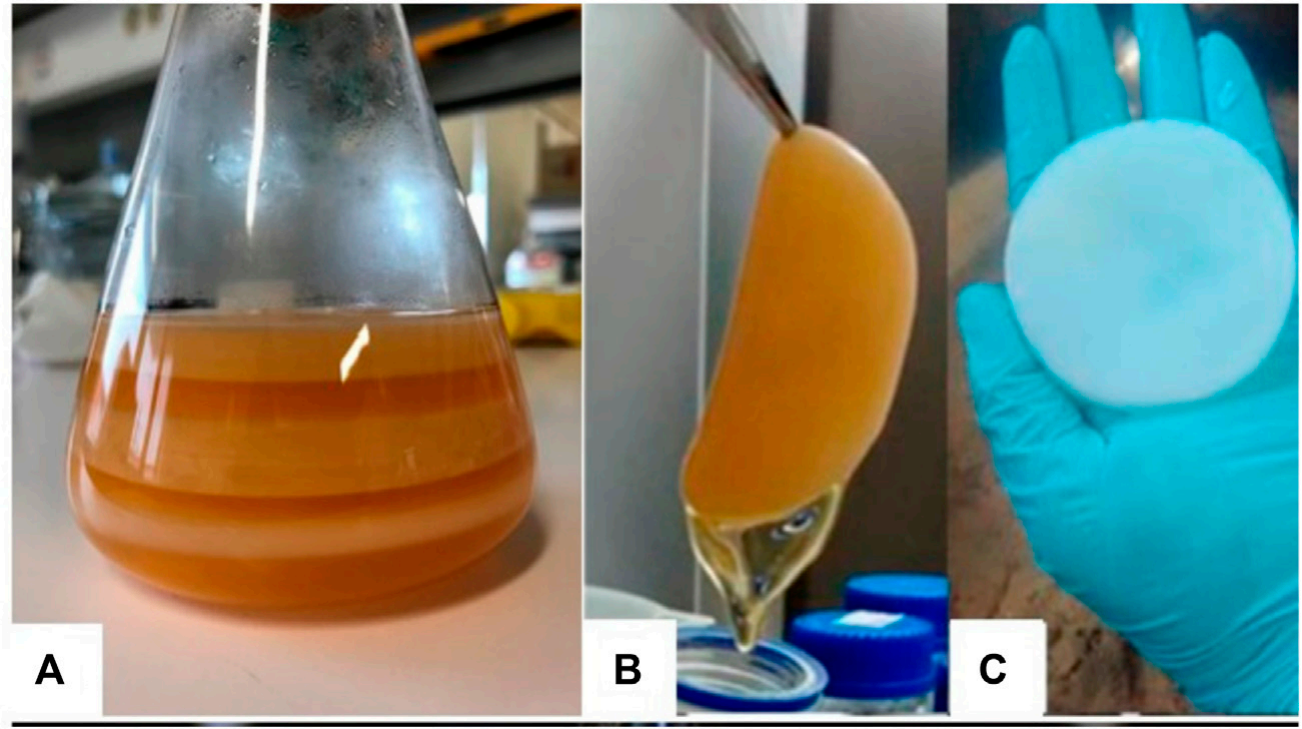
BNC shapes: BNC membranes produced in static fed-batch conditions **(A)**; wet BNC membrane produced in static culture in Hestrin-Schramm (HS) medium, before purification **(B)** and after purification **(C)**. Reprinted with permission from Almeida et al. (2014) under Creative Commons Attribution License (CC BY).

BNC was identified to be a biosynthetic product of *Gluconacetobacter xylinus* specimens by A. J. Brown (1886) in 1886. He discovered that cellulose is the gel-like component that forms in the solution during vinegar fermentation. People have a thorough understanding of the development and structure of BNC as a consequence of meticulous and rigorous investigation for the last few years. This research is crucial in the merger of bio-technological approaches into polysaccharide chemistry and the advancement of cellulosic materials with novel attributes and uses. The biosynthesis of BNC occurs at a cellulose-synthesizing complex in the bacterial cell. It begins with uridine di-phosphate glucose and then adds this intermediate to the developing cellulose molecule’s end. Through terminal complexes (TCs) on the bacteria’s surface, this chain leaves the cell as a basic fibril. The self-orientation of cellulose particles in the presence of a liquid suspension and without complicated components results in a substantially enlarged three-dimensional matrix with evident passageway and pore structures and a moisture content of up to 99%. BNC develops a thin coating at the air-liquid crossing point in stagnant culture using Hestrin and Schramm’s common complex media (Figure 1) (Hestrin and Schramm, 1954). In reality, cellulose self-assembly has long been associated with daily living. The disintegration of natural raw materials (for example wood pulp and cotton linters) is followed by “regeneration,” which includes molecular self-assembly, in the manufacturing of rayon fibres (Wang et al., 2016; Kim et al., 2019). Fabrics made from regenerated cellulose materials have been marketed, demonstrating the practicality of constructed cellulose materials. However, directing the assembly of homopolymers into ordered nanostructures in a regulated manner remains a challenge. “Biosynthesis produces high-purity nanocellulose with a yield of roughly 40% as compared to bacterial strains”. By boiling with a mildly alkaline medium and then washing with water, residual bacteria and medium components can be eliminated. Fink et al. presented a new design of the BNC edifice in the wet condition (Fink et al., 1997). Anhydrous nanofibrils in the 7 × 13 nm range appear to be hydrated entirely and assemble into planar microfibrils with 70–150 nm thickness. This signifies that the water is between these elements and outside the crystalline cellulose nano unit. The amorphous cellulose chain’s shell avoids the surrounding microfibrils, resulting in a microfibril ribbon (ribbon) with a thickness of around 0.5 µm. Bacteria and natural plant cellulose co-occur in two sorts of crystal form, I_α_ (triclinic) and I_β_ (monoclinic). The hydrogen bonding mechanism and the shape of adjacent cellulose chains are the key differences. The I_α_/I_β_ proportion is affected by the cellulose source (Fink et al., 1997). As previously stated, the distinctive and remarkable qualities of the attainable moist nano-fibrillated system underpin the characteristics of bacterial nanocellulose. This nanocellulose is noted for its biocompatibility and plasticity during the culture process, as well as good mechanical qualities, in addition to its greater DP and crystallinity. The thermal expansion coefficient of BNC fiber is low, and it has an improved Young’s modulus and strength properties (Yano and Nakahara, 2004; Yano et al., 2005).

The research on BNC throughout the last 3 decades was examined to acquire a better grasp of the global research status in this discipline. This bibliometric study also serves as a foundation for the establishment of medium and long-term BNC research initiatives. Thus, the analysis synthesized quantitative descriptions of publications retrieved from indexed journals, categories by Web of Science, yearly outputs, and top institutions and leading countries as well as the research trends and hotspots identified through the analyses of paper titles, author keywords, and *KeyWords Plus*. These bibliometric analyses bring together selected bacteria-derived nanocellulose findings and their applications in technical membranes, composites, fuel cells, the food sector, wound dressings, and cosmetic tissues.

## 2 Data and Bibliometric Methods

The data for the present study were obtained from the SCI-EXPANDED of Web of Science in Clarivate Analytics (updated on August 19, 2021). The journal impact factors in 2020 (*IF*_2020_) were presented on June 30, 2021 in Journal Citation Reports (JCR). According to the definition of journal impact factor, it is better to collect the documents published in 2020 from the SCI-EXPANDED after *IF*_2020_ was presented. Although SCI-EXPANDED is created primarily to find and search literature by researchers, it does not present data in a readily available form for bibliometric investigations (Ho, 2018; Ho Y. S. 2021). As a result, data processing is always required for bibliometric studies followed by data collection directly from SCI-EXPANDED. Recently, a big difference was found by using “front page” including the paper title, abstract, and author keywords in the paper (Fu et al., 2012) as a filter in widely bibliometric studies (Ho Y.-S. 2019). *KeyWords Plus* can enhance and supplement title-word and author-keyword indexing by extracting additional search terms from the titles of publications that are mentioned by authors in their bibliographies and footnotes in the ISI (now Clarivate Analytics) database (Garfield, 1990). It was pointed out that the documents that could only be searched using *KeyWords Plus* were unrelated to the topic being searched (Fu and Ho. 2015). After pre-study, words used in SCI-EXPANDED were considered as search keywords including “bacterial nano cellulose”, “bacterial cellulose nano composite”, “bacterial cellulose nano fiber”, “bacterial cellulose nano fibres”, “bacterial cellulose nano fibrils”, “bacterial cellulose nanocomoposite”, “bacterial cellulose nanocomposites”, “bacterial cellulose nanofiber”, “bacterial cellulose nanofibers”, “bacterial cellulose nanofibre”, “bacterial cellulose nanofibres”, “bacterial cellulose nanofibril”, “bacterial cellulose nanofibrils”, “bacterial cellulose nanofibrous”, “bacterial cellulose nanosized fibers”, “bacterial nanocellulose”, and “bacterial nanocelluloses” were searched by the terms of Topic in the SCI-EXPANDED. It results in 642 documents from 2005 to 2020. A total of 39 documents (6.1% of the 642 documents) do not have search keywords on their “front page”. Only 603 documents were defined as bacterial nanocellulose publications. For analysis, these records were imported into a spreadsheet, and additional coding was done manually using Microsoft Excel 2016 (Li and Ho 2008; Ho Y-S. 2021). Moreover, each journal’s impact factor (*IF*_2020_) was acquired from the Journal Citation Reports (JCR) in 2020.

The SCI-EXPANDED is mainly designed for researchers to find literatures but not bibliometric study (Ho, 2018). Thus, it is necessary to have a data treatment when using it for a bibliometric study. The corresponding author is marked as reprint author in SCI-EXPANDED, however, we utilized the term corresponding author in this study (Ho, 2012b). In the case of articles with multiple corresponding authors, only the last corresponding author, institute, and country being designated as the corresponding author information (Ho Y-S. 2019). To have accurate analysis results, affiliations originating from England and Scotland were categorized as being from the United Kingdom (UK).

Three citation indicators were used to analyze the citations received by the articles:

*C*_year_: the total number of citations in a particular year from the Web of Science Core Collection. *C*_2020_ means the number of citations in 2020 (Ho, 2012a).
*TC*_year_: the total number of citations from the Web of Science Core Collection since publication year to the end of the most recent year (Wang and Ho, 2011). In this study, the most recent year is 2020 (*TC*_2020_).
*CPP*_year_: citations per publication (*CPP*_2020_ = *TC*_2020_/*TP*) (Ho, 2012a), *TP* is total number of articles.

## 3 Results and Discussion

### 3.1 Document Type and Language of Publication

In 2004, a connection between document types and citations per publication were proposed (Hsieh et al., 2004). After a decade, the citations per publication were improved by using the citation indicator of *CPP*_year_ which gives more accurate values (Ho and Ho 2015). In 2017, the number of authors per publication (*APP*) was used in the discussion of document types (Monge-Nájera and Ho 2017). Table 1 illustrates the characteristics of seven document types, including 517 articles (86% of the 603 documents) with *APP* of 6.0. The largest number of authors in an article is “A safe and sustainable bacterial cellulose nanofiber separator for lithium rechargeable batteries” (Gwon et al., 2019) published by 34 authors from two institutes in South Korea and one in Japan. The document type of the book chapters had the highest *CPP*_2020_ of 332, which was attributed to the only one highly cited book chapter with a *TC*_2020_ of 100 or more (Ho and Kahn, 2014), by Klemm et al. (Klemm et al., 2006) with a *TC*_2020_ of 332. The *CPP*_2020_ of the reviews was 5.2 times the *CPP*_2020_ of the articles which is much higher than a new research topic of fluorescent carbon nanoparticles with 2.6 times (Yang and Ho 2019). A total of 54 meeting abstracts were published in nine journals mainly in *Abstracts of Papers of the American Chemical Society* (42 meeting abstracts; 78% of 54 meeting abstracts). The only classic publication with *TC*_2020_ of 1,000 or more (Long et al., 2014) in the Bacterial nanocellulose research was a review entitled “Review: Current international research into cellulose nanofibres and nanocomposites” (Eichhorn et al., 2010) with a *TC*_2020_ of 1,581. In addition, five of the top ten most frequently cited publications were reviews in bacterial nanocellulose research. It is worth noting that documents in the Web of Science Core Collection can be split into two document types. For example, five documents were classified as document types of proceedings papers and also articles, thus the sum of the percentages is greater than 100% (Usman and Ho, 2020).

**TABLE 1:** Citations and authors according to document type.

Document type	*TP*	%	*AU*	*APP*	*TC*_2020_	*CPP*_2020_
Article	517	86	3,102	6.0	13,856	27
Meeting abstract	54	9.0	255	4.7	66	1.2
Review	29	4.8	143	4.9	4,018	139
Proceedings paper	5	0.83	28	5.6	64	13
Editorial material	2	0.33	3	1.5	17	8.5
Correction	1	0.17	7	7.0	0	0
Book chapter	1	0.17	7	7.0	332	332

*TP*: number of publications; *AU*: number of authors; *APP*: number of authors per publication; *TC*_2020_: the total number of citations from Web of Science Core Collection since publication year to the end of 2020; *CPP*_2020_: number of citations (*TC*_2020_) per publication (*TP*).

Only 517 articles that included introduction, method and material, results and discussion, and conclusion were chosen for further analysis out of all document categories. One of the most important considerations in bibliometric research as a big data analysis is the language of publishing (Wang and Ho, 2011). English, which accounted for 99% of all articles. Two articles were published in Chinese and one in German.

### 3.2 Characteristics of Publication Outputs

**FIGURE 2:**
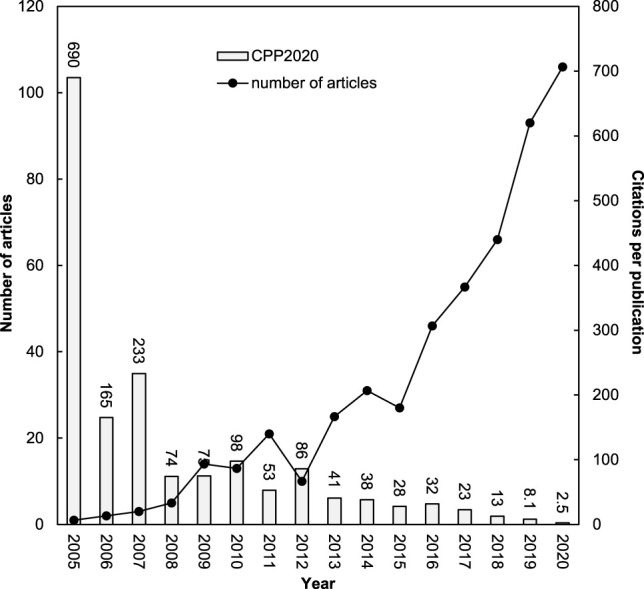
Number of bacterial nanocellulose articles and their citations per publication by year.

A connection between the total annual number of articles (*TP*) and their citations per publication (*CPP*_year_ = *TC*_year_/*TP*) by years has been proposed by Ho to understand publication and their impact trends in a research topic (Ho 2013). Figure 2 depicts the year-by-year distribution of *TP* and their *CPP*_2020_. It has been used as a unique indicator for widely research topics, for example dengue (Ho et al., 2016), metal-organic frameworks (Ho and Fu, 2016), distributed control (Zhai and Ho, 2018), child sexual abuse (Vega-Arce et al., 2019), Fenton oxidation for soil and water remediation (Usman and Ho, 2020), and artificial intelligence (Ho and Wang, 2021). The annual publication outputs increased during the last 6 years sharply. Bacterial nanocellulose have received much attention in recent years especially as a new patch material (Lang et al., 2015). The mean number of *CPP*_2020_ was 27. In 2005, the first article appeared with *TC*_2020_ of 690. Only one highly cited article was published in 2005. In the beginning years, Yano’s group in Kyoto University in Japan, published the main highly cited articles including “Optically transparent composites reinforced with networks of bacterial nanofibers” (Yano et al., 2005) in 2005 with *TC*_2020_ of 690 (rank first) and “Surface modification of bacterial cellulose nanofibers for property enhancement of optically transparent composites: Dependence on acetyl-group DS” (Ifuku et al., 2007) in 2007 with *TC*_2020_ of 251 (rank sixth). In 2005, 2006, and 2007 with one, two, and three articles had the higher *CPP*_2020_ of 690, 165, and 233, respectively, which can be attributed to the articles by Yano’s group in Kyoto University in Japan and Wan’s group in Tianjin University in China.

### 3.3 Web of Science Categories and Journals

**FIGURE 3:**
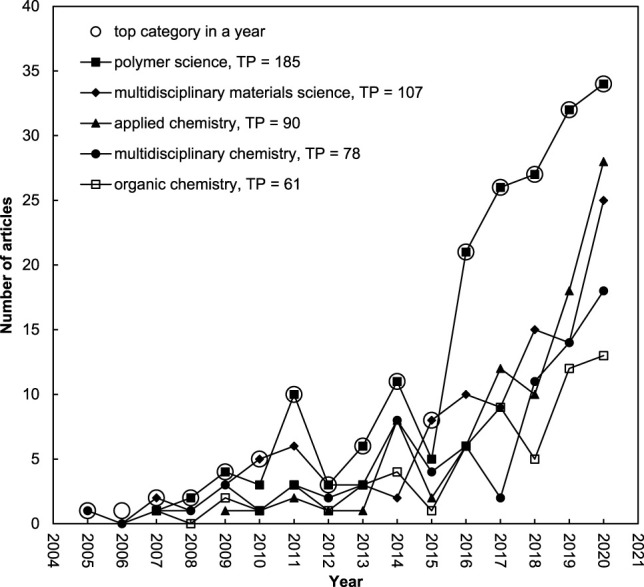
Developments of the top five Web of Science categories with *TP* > 60.

In the year 2020, a total of 9,500 journals was indexed by Journal Citation Reports (JCR) across 178 Web of Science categories in SCI-EXPANDED. Recently, a relationship among number of articles and journals in a Web of Science category as well as number of authors and citations per publication were proposed (Giannoudis et al., 2021). The 518 bacterial nanocellulose-related articles were published in a wide range of 220 journals under 64 Web of Science categories in SCI-EXPANDED. Five of the 518 articles were published in five journals that are not classified in SCI-EXPANDED without journal impact factor in 2020. The top ten Web of Science categories are shown in Table 2. Web of Science category of polymer science was the leading category with 185 articles (36% of 518 articles). Compared to the top 10 categories, bacterial nanocellulose articles with the highest *CPP*_2020_ were published in the category of physical chemistry with *CPP*_2020_ of 63. In 2020, 333 journals were classified in the category of multidisciplinary materials science with 107 articles (21% of 518 articles) ranked second while 22 journals in category of paper and wood materials science with 49 articles ranked ninth. Category of nanoscience and nanotechnology published 51 articles (ranked seventh; 10% of 518 articles) also had a higher *CPP*_2020_ of 59. Average of authors (*APP*) in the category of nanoscience and nanotechnology was 7.3 while polymer science was 5.5. Journals indexed in SCI-EXPANDED can be classified into two or more categories, for example, *Carbohydrate Polymers* was classified in the categories of applied chemistry, organic chemistry, and polymer science thus the sum of percentages could be higher than 100% (Ho and Kahn, 2014). Figure 3 shows the development trends of the top five categories with more than 60 articles. In 2005, the first bacterial nanocellulose article was published in *Advanced Materials* that was classified in the six Web of Science categories: multidisciplinary chemistry, physical chemistry, nanoscience and nanotechnology, multidisciplinary materials science, applied physics, and condensed matter physics. The first article in categories of polymer science and applied chemistry was published in 2007 and 2009, respectively. The category of polymer science published the main part bacterial nanocellulose articles, especially after 2015.

**TABLE 2:** The top 10 productive Web of Science categories.

Web of science category	*TP* (%)	*TC*_2020_	*CPP*_2020_	AU	APP	No. *J*
Polymer science	185 (36)	4,050	22	1,026	5.5	88
Multidisciplinary materials science	107 (21)	4,962	46	675	6.3	333
Applied chemistry	90 (17)	1,867	21	516	5.7	74
Multidisciplinary chemistry	78 (15)	3,239	42	504	6.5	178
Organic chemistry	61 (12)	2,087	34	374	6.1	57
Applied physics	54 (10)	2,349	44	349	6.5	160
Nanoscience and nanotechnology	51 (10)	2,991	59	374	7.3	106
Textiles materials science	51 (10)	838	16	285	5.6	25
Paper and wood materials science	49 (9.5)	829	17	280	5.7	22
Physical chemistry	47 (9.1)	2,942	63	330	7.0	162

*TP*: number of publications; %: percentage of 518 articles; *TC*_2020_: the total number of citations from Web of Science Core Collection since publication year to the end of 2020; *CPP*_2020_: number of citations (*TC*_2020_) per publication (*TP*); *AU*: the total number of authors; *APP*: number of authors per publication; No. *J*: number of journals in a Web of Science category.

The top five most productive journals with more than 10 articles were: *Carbohydrate Polymers* (*IF*_2020_ = 9.381) with 52 articles (10% of 518 articles), *Cellulose* (*IF*_2020_ = 5.044) with 44 articles (8.5%), *International Journal of Biological Macromolecules* (*IF*_2020_ = 6.953) with 23 articles (4.4%), *RSC Advances* (*IF*_2020_ = 3.361) with 12 articles (2.3%), and *Materials Science and Engineering C-Materials for Biological Applications* (*IF*_2020_ = 7.328) with 11 articles (2.1%). According to journal impact factor, *Nature Nanotechnology* with one article, places first with the highest *IF*_2020_ of 39.213, followed by *Energy and Environmental Science* with one article (*IF*_2020_ = 38.532), *Materials Today* with one article (*IF*_2020_ = 31.041), and *Advanced Materials* with four articles (*IF*_2020_ = 30.849).

### 3.4 Publication Performances: Countries and Institutions

**FIGURE 4:**
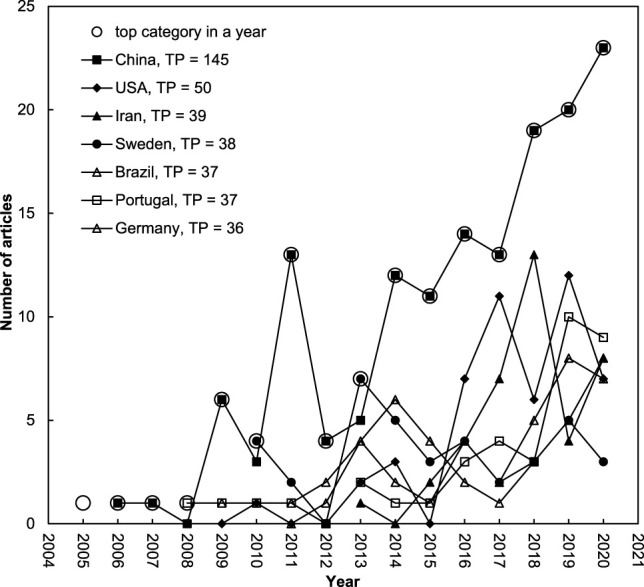
Comparison of development trends among the top seven productive countries with *TP* >.

Of the 518 bacterial nanocellulose articles from 51 different countries, 369 articles (71% of the 518 articles) were single country articles across 31 different countries, while 149 (29%) articles were international collaborations from 48 different countries. The top 10 productive countries are listed in Table 3. Five Asian countries, three European countries, and two American countries made the top ten list of publications. Outside of the top 10, Egypt with four articles ranked 31st was the top productive country in Africa and Australia with three articles ranked 34th. Five publication indicators were used for the comparison of publication performance: total number of articles (*TP*), single-country articles (*IP*), internationally collaborative articles (*CP*), first-author articles (*FP*), and corresponding-author articles (*RP*) (Hsu and Ho, 2014) as well as their *CPP*_2020_ (Fu and Ho, 2018). China dominated among the five publication indicators with a *TP* of 145 articles (28% of 518 articles), an *IP* of 103 articles (28% of 369 single-country articles), a *CP* of 42 articles (28% of 149 internationally collaborative articles), an *FP* of 136 articles (26% of 518 first-author articles), and an *RP* of 126 articles (24% of 518 corresponding-author articles). Compare to the top 10 countries, bacterial nanocellulose articles by Japan had the highest *CPP*_2020_ of *TP*, *FP*, and *RP* with 75, 121, and 121, respectively. Kyoto University in Japan published two of the top ten articles as both first author and corresponding author with *CPP*_2020_ of 609 (rank first) and 251 (rank sixth). Figure 4 shows a comparison of development trends among the top seven leading countries with more than 35 articles. The annual number of articles for a country after 2013 mainly published by China.

**TABLE 3:** Top 10 productive countries.

Country	TP	*TPR* (%)	*TP CPP*_2020_	*IPR* (%)	*CPR* (%)	*FPR* (%)	*FP CPP*_2020_	*RPR* (%)	*RP CPP*_2020_
China	145	1 (28)	28	1 (28)	1 (28)	1 (26)	29	1 (24)	30
United States	50	2 (10)	33	6 (5.1)	2 (21)	4 (5.8)	41	3 (6.8)	38
Iran	39	3 (7.5)	18	2 (8.4)	15 (5.4)	2 (6.9)	19	2 (6.9)	19
Sweden	38	4 (7.3)	43	15 (1.9)	2 (21)	7 (4.1)	61	7 (4.6)	58
Brazil	37	5 (7.1)	16	3 (7.0)	7 (7.4)	3 (6.8)	16	4 (6.6)	17
Portugal	37	5 (7.1)	22	5 (5.4)	4 (11)	4 (5.8)	25	5 (5.8)	25
Germany	36	7 (6.9)	28	4 (5.7)	5 (10)	6 (5.6)	24	6 (5.4)	22
South Korea	25	8 (4.8)	35	7 (4.3)	11 (6.0)	8 (3.9)	39	8 (3.9)	39
Japan	20	9 (3.9)	75	9 (3.0)	11 (6.0)	14 (2.1)	121	15 (2.1)	121
Thailand	20	9 (3.9)	20	9 (3.0)	11 (6.0)	10 (3.1)	7.7	9 (3.1)	7.7

*TP*: total number of articles; *TPR* (%): rank of total number of articles and percentage; *IPR* (%): rank of single country articles and percentage in all single country articles; *CPR* (%): rank of internationally collaborative articles and percentage in all internationally collaborative articles; *FPR* (%): rank of first-author articles and percentage in all first-author articles; *RPR* (%): rank of corresponding-author articles and percentage in all corresponding-author articles; *CPP*_2020_: number of citations (*TC*_2020_) per publication (*TP*).

In the case of performance of institutions, 179 articles (35% of 518 articles) came from a single institution while 339 articles (65%) were collaborative amongst institutions. Only five institutes had 15 articles or more: Donghua University (China) with 41 articles (7.9% of 518 articles), Tianjin University (China) with 25 articles (4.8%), University of Aveiro (Portugal) with 24 articles (4.6%), Federal University of Santa Catarina (Brazil) with 16 articles (3.1%), and Chalmers University of Technology (Sweden) with 15 articles (2.9%). Donghua University also published the most single-institute articles (18 articles; 10%), inter-institutionally articles (23 articles; 6.8%), first-author articles (39 articles; 7.5%), and corresponding-author articles (30 articles; 5.8%). In addition, there is no single-author article in bacterial nanocellulose study.

### 3.5 The Most Frequently Cited Articles

After publication, highly cited publications may or may not have a high impact or visibility (Ho and Kahn, 2014). The number of citations received in the most recent year of 2020 (*C*_2020_) may offer readers extra information about the influence of a highly referenced work today (Ho, 2012a). When the 518 bacterial nanocellulose articles were sorted by *TC*_2020_, a different ranking was generated compared to the ranking obtained from the *C*_2020_ sorting (Table 4). A total of 75 articles (14% of 518 articles) did not receive any citation in the most recent year (*C*_2020_ = 0) and 51 (9.8%) articles had no citations from their publishing year until the end of 2020 (*TC*_2020_ = 0). Moreover, 58% of the top 100 *C*_2020_ articles were also among the top 100 *TC*_2020_ articles. The 518 bacterial nanocellulose articles have been searched with search keywords in their title, abstract, and author keywords. A total of 353 articles (68% of 518 articles); 358 articles (69% of 518 articles with abstract); and 176 articles (42% of 421 articles with author keywords) contained search keywords in their title, abstract, and author keywords, respectively. The title of an article is a label in which supplied reasonable details of the article subjects (Wang et al., 2010). Author keywords were given by authors to offer more information about the main research focused on articles. Articles that contain search keywords in their abstract might less relate to the search topic directly. The top two most frequently cited articles entitled “Optically transparent composites reinforced with networks of bacterial nanofibers” (Yano et al., 2005) and “Making flexible magnetic aerogels and stiff magnetic nanopaper using cellulose nanofibrils as templates” (Olsson et al., 2010) contained search keywords in their abstract only. These articles do not directly relate to bacterial nanocellulose research.

**TABLE 4:** The top 10 most frequently cited articles with search keywords in their title or author keywords.

R (TC_2020_)	*R* (*C*_2020_)	Title	Country	References
3 (331)	8 (40)	All-solid-state flexible supercapacitors fabricated with bacterial nanocellulose papers, carbon nanotubes, and triblock-copolymer ion gels	South Korea	Yu et al. (2012)
5 (252)	39 (18)	Biomimetic synthesis of hydroxyapatite/bacterial cellulose nanocomposites for biomedical applications	China, Canada	Wan et al. (2007)
6 (251)	27 (21)	Surface modification of bacterial cellulose nanofibers for property enhancement of optically transparent composites: Dependence on acetyl-group DS	Japan	Ifuku et al. (2007)
7 (236)	34 (19)	Synthesis and characterization of hydroxyapatite-bacterial cellulose nanocomposites	China	Wan et al. (2006)
10 (190)	15 (26)	Bacterial nanocellulose as a renewable material for biomedical applications	Sweden, Germany	Gatenholm and Klemm (2010)
11 (186)	48 (14)	Synthesis of silver nanoparticles templated by TEMPO-mediated oxidized bacterial cellulose nanofibers	Japan	Ifuku et al. (2009)
12 (151)	29 (20)	Bacterial cellulose nanofiber-supported polyaniline nanocomposites with flake-shaped morphology as supercapacitor electrodes	China	Wang et al. (2012)
13 (141)	24 (22)	Development of transparent bacterial cellulose nanocomposite film as substrate for flexible organic light emitting diode (OLED) display	Canada, Thailand	Ummartyotin et al. (2012)
16 (124)	153 (7)	Proliferation and osteoblastic differentiation of human bone marrow stromal cells on hydroxyapatite/bacterial cellulose nanocomposite scaffolds	China	Fang et al. (2009)
17 (122)	12 (29)	Active wound dressings based on bacterial nanocellulose as drug delivery system for octenidine	Germany	Moritz et al. (2014)

*TC*_2020_: the total number of citations from Web of Science Core Collection since publication year to the end of 2020; *C*_2020_: the number of citations of an article in 2020 only; *R*: ranking in 518 bacterial nanocellulose articles.

**FIGURE 5:**
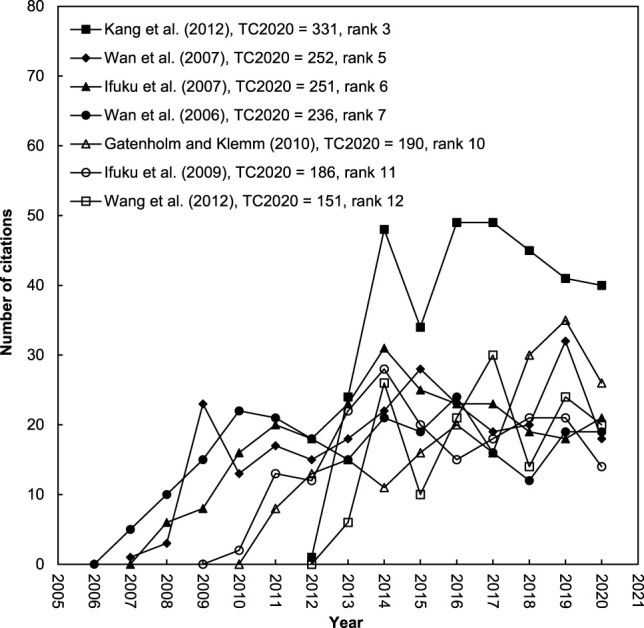
The citation histories of the top seven most frequently cited articles with search keywords in their title or author keywords (*TC*_2020_ > 150).

Figure 5 shows the citation trends of the top seven most often cited articles with search keywords in the title or author keywords. Article by Ifuku et al. (2009) ranked 11th on *TC*_2020_ with 186 but ranked 48th on *C*_2020_ with 14. Similarly, an article by Wan et al. (2007) ranked fifth on *TC*_2020_ with 252 but ranked 39th on *C*_2020_ with 18. Article entitled “All-solid-state flexible supercapacitors fabricated with bacterial nanocellulose papers, carbon nanotubes, and triblock-copolymer ion gels” by Kang et al. (2012) from South Korea ranked top ten in both *TC*_2020_ and *C*_2020_ with 331 (rank third) and 40 (rank eighth), respectively.

### 3.6 Research Focuses

Analysis of used words in publication titles, abstracts, author keywords, and *KeyWords Plus* was proposed for the research focuses (Zhang et al., 2010). In SCI-EXPANDED, 421 (81% of 518 publications) and 511 (99%) bacterial nanocellulose articles contained author keywords and *KeyWords Plus* information, respectively. Table 5 shows the top 20 most used words provided in each of the article titles, author keywords, and *KeyWords Plus*. Except for search keywords, a total of 51 articles (10% of 518 articles), 22 articles (5.2% of 421 articles with author keyword information), and 51 articles (10% of 511 articles with *KeyWords Plus* information) contained “properties”, “mechanical properties”, and “mechanical-properties” as the most used words, respectively. The results showed that “mechanical properties” were the main research focus in BNC study for different applications as shown in Table 6.

**TABLE 5:** The top 20 most used words in title, author keywords, and *KeyWords Plus*.

Word in title	*TP*	*R* (%)	Author keywords	*TP*	*R* (%)	*KeyWords Plus*	*TP*	*R* (%)
Bacterial	392	1 (76)	Bacterial cellulose	156	1 (37)	Cellulose	94	1 (18)
Cellulose	247	2 (48)	Bacterial nanocellulose	123	2 (29)	Composites	72	2 (14)
Nanocellulose	180	3 (35)	Nanocomposites	27	3 (6.4)	Bacterial cellulose	61	3 (12)
Nanofibers	90	4 (17)	Mechanical properties	22	4 (5.2)	Nanoparticles	56	4 (11)
Nanocomposites	52	5 (10)	Nanocomposite	22	4 (5.2)	Mechanical-properties	51	5 (10)
Properties	51	6 (10)	Bacterial cellulose nanofibers	18	6 (4.3)	Nanocomposites	46	6 (9)
Novel	44	7 (8.5)	Wound dressing	14	7 (3.3)	Acetobacter-xylinum	36	7 (7)
Nanocomposite	42	8 (8.1)	Nanocellulose	12	8 (2.9)	Membranes	34	8 (6.7)
Composites	30	9 (5.8)	Tissue engineering	12	8 (2.9)	Films	33	9 (6.5)
Flexible	30	9 (5.8)	Nanofiber	10	10 (2.4)	Microbial cellulose	32	10 (6.3)
Production	29	11 (5.6)	Polypyrrole	10	10 (2.4)	Cellulose production	30	11 (5.9)
Preparation	26	12 (5.0)	Antimicrobial activity	9	12 (2.1)	Fibers	30	11 (5.9)
Films	25	13 (4.8)	Wound healing	9	12 (2.1)	Fabrication	26	13 (5.1)
Nanofiber	25	13 (4.8)	adsorption	8	14 (1.9)	Gluconacetobacter-xylinus	26	13 (5.1)
Situ	25	13 (4.8)	Biocompatibility	8	14 (1.9)	Composite	25	15 (4.9)
Poly	23	16 (4.4)	Chitosan	8	14 (1.9)	Nanofibers	25	15 (4.9)
Wound	23	16 (4.4)	Gluconacetobacter xylinus	8	14 (1.9)	Acid	24	17 (4.7)
Characterization	22	18 (4.2)	HHydrogel	7	18 (1.7)	Behavior	24	17 (4.7)
Mechanical	22	18 (4.2)	Hydroxyapatite	7	18 (1.7)	Biosynthesis	24	17 (4.7)
Membranes	22	18 (4.2)	Immobilization	7	18 (1.7)	Adsorption	23	20 (4.5)
Nanoparticles	22	18 (4.2)	Laccase	7	18 (1.7)	*in vitro*	23	20 (4.5)
Surface	22	18 (4.2)	Nanofibers	7	18 (1.7)	—	—	—
Synthesis	22	18 (4.2)				—	—	—

*TP*: number of publications including the word in their title, author keywords, and *KeyWords Plus*; *R* (%): rank of words used and percentage in title, author keywords, and *KeyWords Plus*.

**TABLE 6:** Areas of bacterial nanocellulose applications.

Sectors	Uses
Cosmetic industries	Cosmetics preservative of emulsions such as creams, conditioners, lotions, etc.
Textile industries	Sports and leisure apparel, shelters and camp out utensils
Mining and processing plant sectors	Wipers to accumulate dripping oil, constituents for toxic absorption
Waste treatment plant	Recycling of natural resources
Manure refinement process	Municipal sewage refinement, extreme filtration of water
Communication industries	Headset and speaker diaphragms
Food industry	Fit for human consumption cellulose (nata de coco)
High grade paper	Substitution of wood
Medicine/biomedical applications	Artificial skin, medicine delivery, dressing of wound, burns, dental implants, etc.
Research labs	Culture medium for tissue engineering, chromatography, immobilization of protein
Electronic devices	Biosensors, capacitors, displays
Energy sectors	Membrane fuel cell containing palladium

#### 3.6.1 Food Industry Applications

**FIGURE 6:**
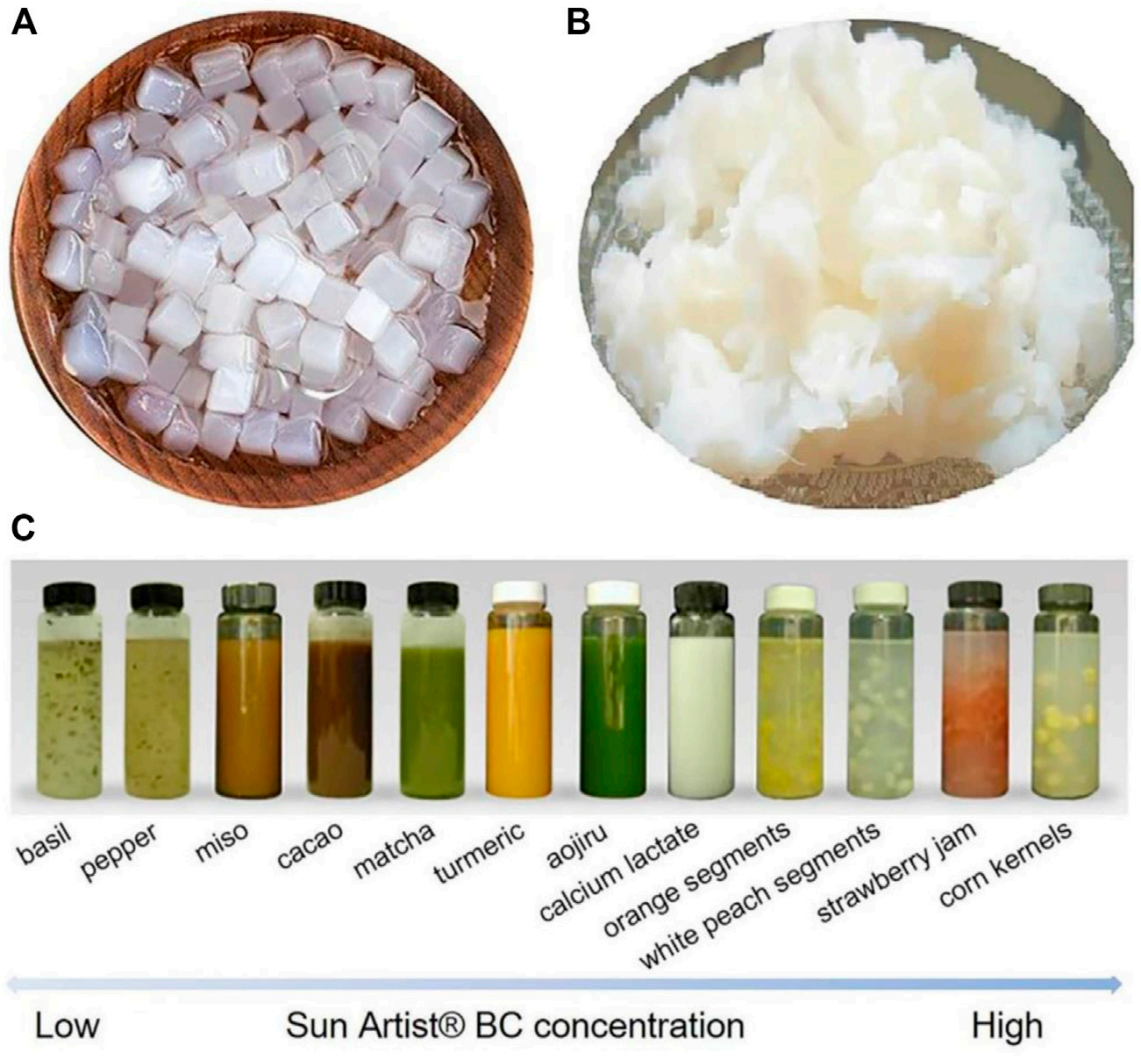
Utilization of BNC granules as food additives; **(A)** a static fermentation and cutting procedure produces the cube-shaped BNC, and **(B)** a two-step compression approach produces the compressed BNC. **(C)** Sun Artist® BNC is a high-stability suspension agent for a variety of solid food products. Reprinted with permission from Zhong (2020) under Creative Commons Attribution License (CC BY).

Since 1992, BC has been regarded by the FDA as a “generally recognized as safe (GRAS)” food additive (Shi et al., 2014). Conventional desserts, limited cholesterol diets, vegetarian meals, food/beverage additions, and packaging materials for food could all benefit from it (Azeredo et al., 2019). One of the original applications of nanocellulose was as a food additive (Figure 6); nevertheless, the cost of generating it is prohibitively expensive, making commercialization impractical. Nonetheless, over time, research has been able to improve the material’s production process, lower prices, and make it possible in this sector (Gómez H et al., 2016). The form and function of BNC in commercial applications are dependent on a technique known as fermentation. BNC obtained through fixed fermentation with a film which looks like jelly is mostly utilized as a raw material for desserts and ingredients, whereas BNC attained through disconcerted fermentation having characteristics termed as hydrocolloid is employed as a thickening and suspending ingredient in liquid refreshment. Cellulase is not found in the human body. Accordingly, BNC is expelled through feces rather than being metabolized and consumed in the gastrointestinal tract (Fontana et al., 2017). The first use of BC in food dates back to the 1960s and 1970s in the Philippines (Iguchi et al., 2000). Nata de coco (NDC) is noted for having a great and uncontaminated content of fiber, as well as being low in calories and cholesterol (Ullah et al., 2016). As a result, it is similar to dietary fiber found in everyday foods, and it is beneficial for human health by lowering the danger of long-lasting sicknesses including diabetes, obesity, and cardiovascular disease (Anderson et al., 2009). Traditionally, NDC is sliced into dices and marinated in a variety of flavors. Furthermore, by altering the fermentation conditions and/or medium formula during the fermentation process, BC of various shapes, textures, and flavors can be produced, greatly expanding the types of BC products available (Gatenholm and Klemm, 2010). It has long been used as a dessert because of its levelheaded and flat texture, crispy, and succulent savor. It is also used as a food additive to give food a new texture and flavor (Shi et al., 2014). NDC is now available in wide variety of meals, including beverages, yogurt, pastries, sausage rolls, and salads. In the beginning, it was popular in the Philippines, and subsequently in Japan and additional nations of Asia during 1990s (Dourado et al., 2016). BC, on the other hand, is employed in the food sector as a beneficial addition. Many beverages and watery foods,—for example dairy drinks, cocoa, oatmeal, and soy milk, have granular components that must be suspended. To suspend particles, thickening agents and surfactants including guar gum, pectin, CMC, and soya milk are commonly added to liquid solutions (Dourado et al., 2016). These formulations, on the other hand, have a deprived dispersion constancy and are frequently plagued by transparency distortion and segment segregation (John McArthur Swazey and Madison, 2011). Furthermore, the excessive viscosity imparts an undesirable flavor to the product. As a result, a new type of suspending agent is required, one with great dispersion stability and low viscosity. It was discovered that the BC particles formed by stirred fermentation have good properties and can effectively perform the role of suspended particles under these conditions (Zhong, 2020). BC seems to have a distinctive structure consisting of a woven 3D network of nanofibers that allows it to suspend low-viscosity insoluble particles very well (Swazey, 2014). Even when there are a lot of surfactants and thickening agents, it can perform well as a suspension agent at low concentrations. Furthermore, BNC is not charged and is suspended rather than dissolved in the solution. This function reduces BNC’s impact on environmental variables including acidity and ionic strength (Swazey, 2014). As a result, BNC preserves its ability to suspend in a wide pH range and has a high salt tolerance. BNC has exceptional enzyme resistance due to its strong crystallinity (Torres et al., 2019). Eventually, BNC maintains its suspension stability at temperatures as high as 80°C (San-Ei Gen F.F.I., Inc., 2020). BNC is a cost effective and irreplaceable particle suspension because of these benefits. Several businesses have employed BNC as a suspending agent in agitated fermentation for commercial food applications. Initially, CPKelco sold BNC as a wet cake, which typically contained 10–20% solids and rest contains water (John et al., 2013). Sorbic acid is also used in this recipe to keep mold away (John et al., 2013). To reestablish the dispersed form in the solution, high-speed shear is used to activate the wet cake. AxCel® PX, AxCel®CG-PX, AxCel®PG, CellulonTMPX, and a series named after “K” have also been created by CPKelco in a dry powder form (John McArthur Swazey and Madison, 2011). BNC is usually combined with one or more surfacting and/or thickening agents, for example guar gum, CMC, pectin, carrageenan, and so on, in these dry powder forms (Swazey, 2014). When BNC comes into contact with water, these additions help it to revert to its scattered state. Auxiliaries, on the other hand, have an unfavorable effect on the liquid medium constancy of BNC because polymers are usually charged. Once dry particles are mixed with low acidity or solutions having good ionic strength, the charged polymers could become immiscible and lose their function, resulting in an unstable or opaque suspension system (John McArthur Swazey and Madison, 2011). To improve the shelf life of the packaging food components, Atta et al. created an edible and bioactive food packaging film that incorporates yeast combined with bacterial cellulose (BC) with carboxymethyl cellulose and glycerine (Atta et al., 2021).

#### 3.6.2 Use in Personal Care Materials and Domestic Chemicals

**FIGURE 7:**
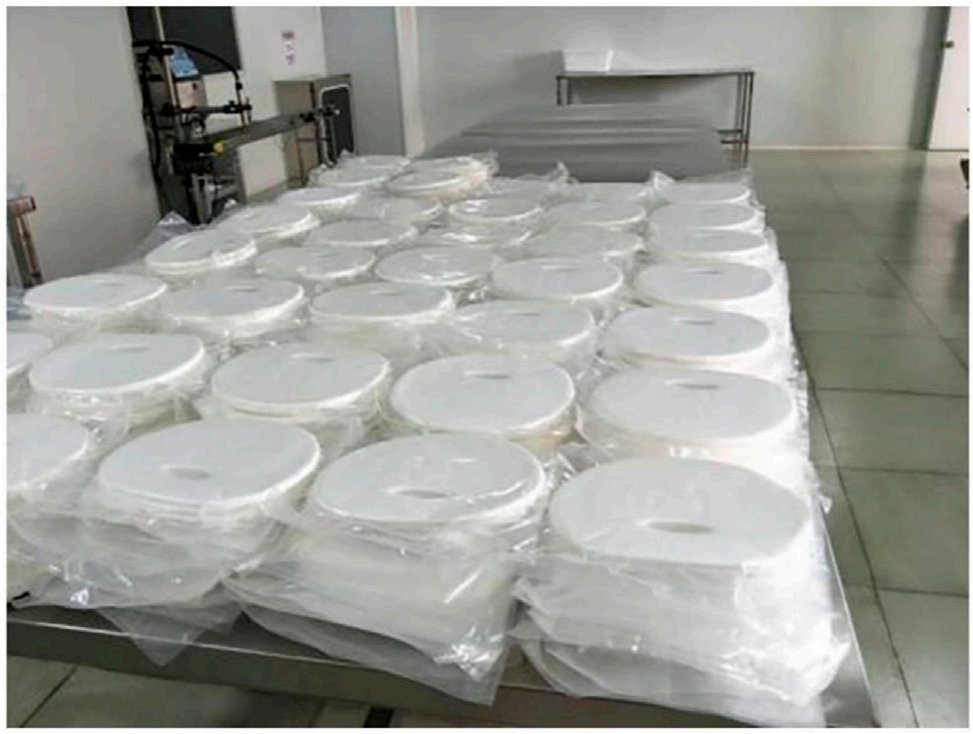
Face masks made from BNC-based raw material. Reprinted with permission from Zhong (2020) under Creative Commons Attribution License (CC BY).

Individual care goods and domestic chemicals are BNC’s second largest utilization sector (Bianchet et al., 2020). Non-toxic and biodegradable component materials should be used in skincare products. As a result, people prefer natural products that are high in purity and reliability. BNC is a natural substance made from bacterial fermentation that has been shown to be extremely biocompatible (Roman, 2015). BNC film obtained from static fermentation has been used as a basic material for masks in personal care products. BNC-containing masks have a superior water holding capacity than non-woven cellulose or silk masks, and provide good cooling and smoothness because to their nano-level 3D mesh network (Amnuaikit et al., 2011; Pacheco et al., 2018). Furthermore, BNC’s very porous microstructure allows it to be loaded with a variety of nutrients, including medicinal compounds (Chantereau et al., 2020). The BNC film’s porosity microstructure also allows it to manage the release of these embedding agents (Numata et al., 2015; Perugini et al., 2018). Because of this function, the mask made of BNC may also be utilized for cosmeceuticals and the cure of moderate skin problems (Almeida et al., 2014; Morais et al., 2019). Furthermore, BNC generated from stirred fermentation can be utilized as a suspending agent, similar to how it is used in food, ensuring that ornamental beads, encapsulated spices, and encapsulated enzymes are well suspended (Swazey, 2014). CPKelco and Kusano Sakko both have aqueous laundry, washing powder, and personal grooming products (Figure 7) on the market (Rajwade et al., 2015; Anton-Sales et al., 2019).

#### 3.6.3 Application in Biomedical Areas

**FIGURE 8:**
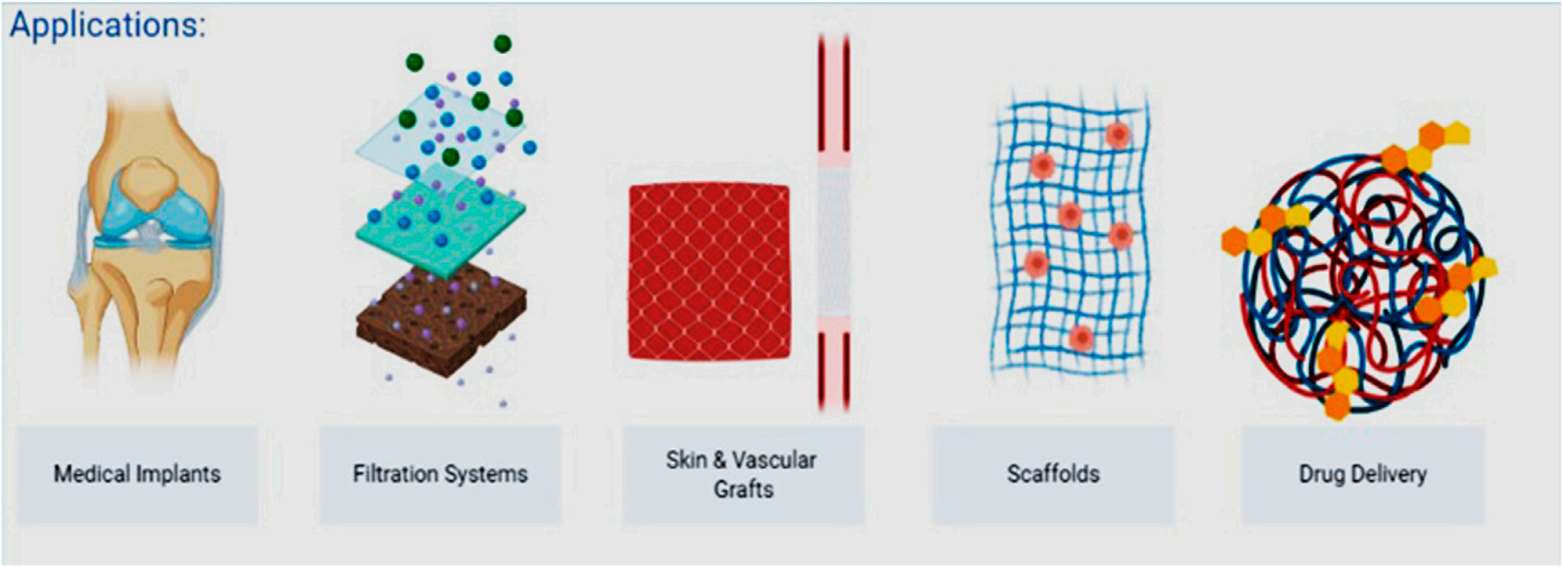
An overview of BNC implants used in tissue restoration and regrowth reprinted with permission from Swingler et al. (2021) under Creative Commons Attribution License (CC BY).

**FIGURE 9:**
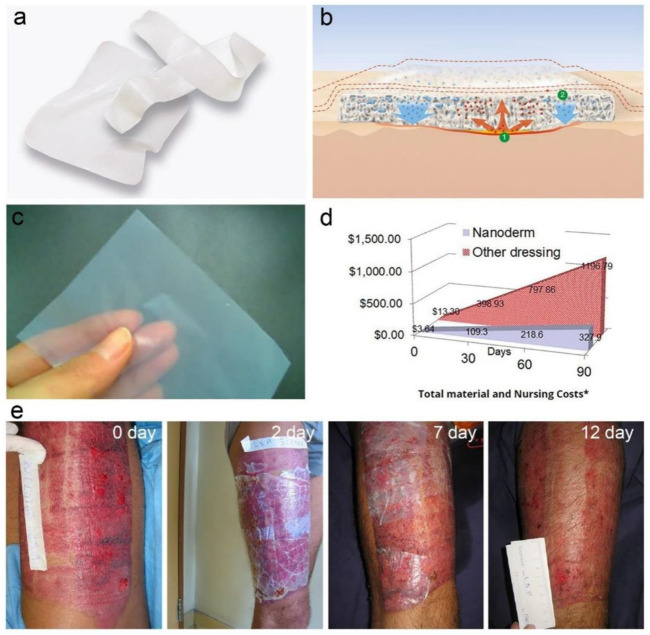
Wound dressing made from BNC; **(A)** the wet wound dressing Suprasorb® X is made up of 1.5–4.3% BNC and other balanced water, **(B)** demonstration of the ability of Suprasorb® X wound dressing to absorb wound exudate and transport water to regions with low exudate. **(C)** NanodermTM, a flexible and transparent dry wound dressing. **(D)** The total cost of NanodermTM wound dressing versus traditional wound dressing in terms of materials and care. **(E)** The skin donor location is dressed with NanodermTM wound dressing. The initial donor location, as well as 2, 7, and 12 days following therapy, are shown in the photographs from left to right. Reprinted with permission from Zhong (2020) under Creative Commons Attribution License (CC BY).

Dressings of injury, imitation skin, dental transplants, medication delivery, hemostatic constituents, vascular grafts, tissue engineering, biosensors, and diagnostics are just a few of the biomedical applications where BNC holds a lot of promise (Figure 8) (Rajwade et al., 2015; Anton-Sales et al., 2019; Carvalho et al., 2019). All biomedical applications necessitate BNC because of its high purity and biocompatibility (Zheng et al., 2020). The endotoxin in BNC is likewise highly controlled, with less than twenty endotoxin units, which fulfills the FDA’s *in vivo* standards (Petersen and Gatenholm, 2011). BNC also offers a one-of-a-kind 3D mesh network with a variety of benefits, including a huge surface area, high water retention capacity, superior liquid/gas penetration, exceptional mechanical qualities, and transparency (Thomas, 2008; Sulaeva et al., 2015). These distinguish BNC as a distinct material that can demonstrate its excellence in biomedical applications. Wound dressing devices based on BNC have been successfully commercialized, and various items relating to medicine delivery, contact lenses, blood vessel transplantation, and tympanic membrane transplantation are also in the works (Coelho et al., 2019). The skin is the human body’s biggest organ. It defends us against microorganisms, keeps our bodies in a state of homeostasis, and controls our body temperature and senses (Zhang et al., 2019). These functions will be lost in diseased skin, resulting in catastrophic consequences. There are a variety of causes for traumatic skin loss, including internal issues like vascular disease, cardiovascular disease, and diabetes, as well as external events like casualties, scalds, as well as surgery (Vogelnest, 2017). Traditional surgical treatment, followed by the use of wound dressings to completely cover the skin lesions, are standard treatment approaches for skin regeneration. The optimum wound dressing will keep the site moist, eliminate exudate, permit sweat and oxygen interchange, limit electrolyte and protein depletion, prevent infection, relieve hurt, and speed wound restorative (Portela et al., 2019). Traditional wound dressings—for example gauze and manmade materials, on the other hand, are unable to achieve these needs. Because of its outstanding moisture control, excellent wet tensile strength, breathability, adjustability, optical transmittance, and strong biodegradability, BNC was originally employed as a wound dressing (Madkour et al., 2019). BNC was discovered to have a number of additional benefits after real application, including removing exudate while enabling sweat and air interchange, lowering agony and electrolyte and protein depletion, preventing infection, and speeding wound closure (Abeer et al., 2014). These are important considerations. BNC’s qualities helped it gain a foothold in the wound dressing equipment market. Consequently, a chain of BNC centered wound bandages are marketed for instance NanodermTM (ND), Bionext®, Membracell®, Suprasorb®X (SX), and others (Figure 9) (Abeer et al., 2014). BNC-based wound dressings are more effective than gauze or manmade materials in the treatment of arteriovenous ulcers, diabetic ulcers, burns, wounds, skin implants, cuts, and other conditions (Portela et al., 2019). Biomaterials made of nanocellulose have qualities that are comparable to those of natural tissues, making them ideal for cell adhesion and proliferation. Nanocrystal suspensions can be employed as a culture environment for this purpose (Liu et al., 2014). An aqueous solution on nanocrystals at a regulated concentration can produce a hydrogel, according to Bhattacharya et al. (2012), which can be utilized as a support for establishing a suitable environment and possesses mechanical qualities that are advantageous to cell diversity and development. Drug carriers made of nanocellulose-based hydrogels can be utilized to control the rate of drug release and successive levels in the body (Mishra et al., 2018). Wet or dry film wound dressings based on BNC are available on the market. SX wound bandage, for example, is a moist film made of 1.5–4.3% BNC and further balanced water. Water balance can be achieved with SX wound dressing in long-lasting wound cuts. It absorbs 20–200% of the weight of the discharging wound lesion’s liquid exudate and can transmit water to more than 75% of the dry or necrotic wound lesion. Axcelon Dermacare Inc., (ADI) created the ND wound covering, which is a dry BNC layer. ND wound covering is a semi-translucent BNC membrane having a wideness on an average of 0.05 mm. The dried film can be simply kept without fear of contamination or mold and bacteria growth. It is similar to wet dressings in terms of functions and benefits, and it is frequently employed in the treatment of skin problems. ND wound dressings, for example, are used to treat skin donor sites and can effectively preserve lesions of skin loss while also assisting skin redevelopment around 12 days. They also marketed NanodermTM Ag, a new improved wound dressing for the treatment of infected wounds. Silver nanoparticles that have been chemically reduced are attached to BNC and gradually release silver ions to exert antibacterial properties. NanodermTM Ag is also antimicrobial, which can help you go longer between dressing changes. Furthermore, ND products are less expensive than conventional wound dressings (Axcelon Dermacare Inc, 2020). BNC has a lot of potential in various biomedical applications besides commercial wound dressings (Picheth et al., 2017; Pacheco et al., 2018). Kusano Sakko Inc. (KSI) revealed that they are using BNC as a medicine transporter to transport anticancer medicines, and they have discovered that BNC can help with regulated drug release. Furthermore, they want to employ BNC as a basic substance for medicines in order to increase medication distribution. ADI has stated that they are working on an oral vaccination that will use BNC as a medication transporter to keep the serum active throughout the stomach’s transit. ADI has announced that it is working on contact lenses, vascular grafts, and prosthetic tympanic membranes, among other BNC-based medical products (Axcelon Dermacare Inc, 2020). BNC has already been investigated for use in the production of vascular grafts (Pacheco et al., 2018). Its strong biocompatibility and outstanding wet mechanical strength make it an attractive contender for vascular implants. Jenpolymer Materials Ltd. and Co. innovated vascular implants for coronary artery bypass surgery under the Basyc trademark (Schumann et al., 2009; Picheth et al., 2017). Innovatec and Axcelon Dermacare Inc., are two other firms that have announced their BNC vascular graft-based device tubing (Czaja et al., 2007; Portela et al., 2019). Furthermore, Mao, Lin et al. devised hydrogel by *in-situ* synthesis of selenium nanoparticles coated with BC/gelatin which represents increased antibacterial, antioxidant, and anti-inflammatory properties to aid skin wound healing (Mao et al., 2021). Figures 8, 9 exhibit typical applications of BNC in biomedical field.

#### 3.6.4 Textile Application

**FIGURE 10:**
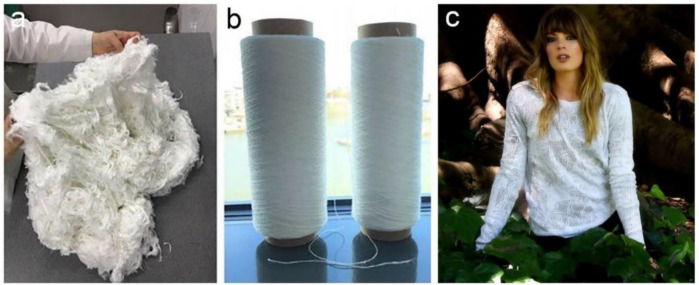
Nanollose makes NullarborTM fibre **(A)**, yarn **(B)**, garments from tree-free viscose rayon fibre **(C)**. Reprinted with permission from Zhong (2020) under Creative Commons Attribution License (CC BY).

BNC is as well employed as an origin of plant free rayon and fabrics as a raw material (Huang et al., 2014). Owing to their nondegradable attribute, the widespread usage of petroleum-based synthetic fibers—for example nylon, polypropylene, and polyester has resulted in major ecological contamination issues (Wei and Zimmermann, 2017). Wood pulp and cotton pulp are commonly used to make plant-based regenerated fibers like rayon and cupramonium. Despite the fact that they are biodegradable, the pulping procedure takes a lot of energy and pollutes the environment due to the usage of enormous amounts of chemicals. Nanollose Ltd. is an Australian technology firm that has pioneered the transformation of BNC into environmentally benign fibers for textile (Figure 10) as well as other manufacturing uses water (Nanollose Ltd, 2020). BNC is easier to purify than plant cellulose, resulting in a lower impact on the environment. They were able to convert BNC into viscose rayon fiber, which can be used instead of plant fiber. During 2018, Nanollose teamed up with PT Supra Natami Utama of Indonesia and build a plant to create BNC suitable for textile uses from coconut water fermentation. Nanollose develops its own method to convert BNC into Nullarbor^TM^ fiber, a viscose rayon fiber that is spun into yarns, fabrics, and garments. Nullarbor^TM^ fiber’s future market can be expanded to include all classic rayon uses, such as shirts, sports, costumes, sports and recreation, and home items (Nanollose Ltd, 2020). They claim that plant-free rayon fibers produced by them have a number of benefits over plant-based fibers in a variety of ways. Rather than using wood pulp extracted through tough chemical procedures, they use industrial waste as a source of cellulose. In terms of time and land needs, fermentation is likewise more efficient than plant growth. Eventually, the BNC fermentation method uses less energy and water in the production of rayon. In conclusion, it appears to be a promising route for BNC applications, offering ecological alternatives to rayon fibers derived from plants.

#### 3.6.5 Application in Composite Materials

Polymers can be added to the culture medium throughout the fermentation procedure to create BNC with variable physical and chemical properties (Chen et al., 2011). Chemical modification of plant cellulose to produce cellulose nanofibers with various chemical and physical attributes is normally a time-consuming and difficult process (Thomas et al., 2018). CMC and xyloglucan, for example, have been found in studies to impact not just the crystal structure and agglomeration of BNC, but also its surface chemistry and solubility. This allows for the creation of BNC with various properties. Under the brand name Fibnano, KSI has productively created a distinctive BNC (Tajima et al., 2017). It consists of CMC, hydroxyethyl cellulose (HEC), and hydroxypropyl cellulose (HPC) (Zhong, 2020). CMC ornamented BNC, HEC adorned BNC, and HPC decked BNC are thinner nanofibers than natural BNC, according to their findings. These modified fibers have an average diameter of 20–50 nm, which is tinier than normal BNC (Tajima et al., 2017). CMC ornamented BNC and HEC adorned BNC, as BNC, shows hydrophilicity and spread effectively in water. HPC decked BNC, on the other hand, is amphiphilic and may be distributed in mutually water and organic solvents (Tajima et al., 2017). HPC decked BNC can also be mixed with poly-methyl methacrylate additive to make a highly dispersible resin (PMMA). The PMMA composite resin retains transparency when 1 wt% of HPC decked BNC is added, whereas the resin made of 1 wt% CMC ornamented BNC appears cloudy (Tajima et al., 2017). The mechanical qualities of PMMA resin implanted with HPC decked BNC have also improved dramatically, with a 15% improvement in tensile strength (Tajima et al., 2017). BNC may be manufactured into numerous varieties by addition of polymers, nanoparticles, and additional substances to the fermentation process, and then raw materials having diverse physical and chemical properties can be further developed to reach a larger range of uses, according to the business case.

#### 3.6.6 Applications in Electronic Devices

The need for devices with energy storage capabilities has been expanding as electronic technology progresses, and these devices are built in an easy-to-access and small manner without sacrificing functionality. Since the decrease of non-renewable reserves, the use of biodegradable polymers has become increasingly popular (Kotatha et al., 2018). In a solid electric double layer capacitor (EDLC), BNC covered with chitosan and alginate deposits were used to make a new gel electrolyte comprising 1-ethyl-3-methylimidazolium tetrafluoroborate and a divider. Inoculation of bacterial cellulose, oxidation in KIO_4_ solution, and coating of chitosan layers substituting with alginate layers yield these gel electrolytes. The gel electrolyte has been designed specifically for utilization in dual-layer free of solvent capacitors (Kotatha et al., 2018).

Polyaniline (PA) was formed on the surfaces of BNC and Graphene nanosheets (GN) simultaneously, resulting in a ternary composite material with increased conductivity on the BNC/GN nanocomposite, according to Wan et al. (2018). The effects of treating conditions like reaction time, temperature on the morphological features, electrical properties, and mechanical characteristics of BNC/GN/PA were evaluated. The findings suggest that deposition of PA in a BNC/GN nanocomposite could be a hopeful way for making a BNC/GN/PA conducting nanocomposite for electro - magnetic protection and flexible electrodes. Xie et al. (2018) developed a biocompatible composite with dual conductive electron and ionic capacity made of bacterial cellulose and flexible conductive polydopamine. It is made by doing *in-situ* self-polymerization of dopamine in bacterial cellulose at a neutral pH. It performs well and can be employed in bio-electrodes and wearable medical devices as a flexible biosensor.

An electroactive hydrogel with BNC was successfully created through cellulose breakdown and physical and chemical crosslinking. The prepared hydrogel with 3D compact nanostructured with showed thermal stability, mechanical characteristics, recoverability, and water absorption. The electroactive hydrogel displayed good biocompatibility and could be advantageous for NIH_3_T_3_ cell growth, according to the *in vitro* biological evaluation (Wang et al., 2020).

## 4 Conclusion

Several key aspects about global research trends of BNC were presented by analyzing bibliometric information available in title, keywords, Keywords plus, author keywords, and author performance of highly cited articles from 2005 to 2020 published in SCI-EXPANDED. The analyses using supporting words in the title, author, keywords, abstract, and *KeyWords Plus* were analyzed to locate research focus and research trends. BNC researches increased sharply during this period. Many studies in 64 Web of Science categories including polymer science, multidisciplinary material science, applied chemistry, multidisciplinary chemistry, and textiles material science have been performed to develop an ideal solution for improving mechanical strength of BNC for various applications. BNC articles were published mainly in the category of polymer science in the last 5 years. Articles are published in English only with three exceptions; two articles were appeared in Chinese and one in German. Although a single language was dominant in BNC research, the research works were moderately spread across the globe (51 different countries) with international collaborations from 48 different countries. Five Asian countries made place in the top 10 countries in this field followed by European countries (two countries). Two Chinese universities led the publication of BNC research followed by a university from Portugal, whereas Kyoto University in Japan had the highest *CPP*_2020_ (609). Six important future research hotspots of BNC have been identified. BNC research is moving forwards with a goal of mechanical strength improvement for their applications in personal care materials to electronic devices. However, other review works (Islam et al., 2017; Ul-Islam et al., 2020) concluded otherwise such as yield improvement of BNC, production cost minimization, and biomedical applications were identified as the main focus area of BNC research. Numerous attempts have been done over the last 15 years to isolate strains that produce cellulose with high efficiency, and many sources have been demonstrated to increase BNC output. Nevertheless, because the majority of studies used *Gluconacetobacter xylinus*, more research is needed to see if alternative bacterial strains are more productive. Furthermore, BNC’s yield and productivity have grown as a result of study in the field of cost-effectiveness of the culture media. Since BNC is a substance with significant industrial value and used in a wide range of industries, additional work is needed to forge this biotechnological substance a viable and economically feasible product. In short, while various findings have looked into the utilization of BNC, further research is desirable to determine the practicality of biotechnological manufacturing, especially in regarding the cultural media’s cost-effectiveness. As a result, more BNC applications are permitted, particularly in nanotechnology (e.g., nanoparticles for medication delivery, beauty products, and food products) and the environment.
